# Acute coronary syndrome in young adults from a Malaysian tertiary care centre

**DOI:** 10.12669/pjms.324.9689

**Published:** 2016

**Authors:** Fan Kee Hoo, Yoke Loong Foo, Sazlyna Mohd Sazlly Lim, Siew Mooi Ching, Yang Liang Boo

**Affiliations:** 1 Dr. Fan Kee Hoo, MRCP Faculty of Medicine and Health Sciences, Universiti Putra Malaysia, Serdang, Malaysia; 2 Dr. Yoke Loong Foo, MRCP Faculty of Medicine and Health Sciences, Universiti Putra Malaysia, Serdang, Malaysia; 3 Dr. Sazlyna Mohd Sazlly Lim, MRCP Faculty of Medicine and Health Sciences, Universiti Putra Malaysia, Serdang, Malaysia; 4 Dr. Siew Mooi Ching, MMed(FM). Faculty of Medicine and Health Sciences, Universiti Putra Malaysia, Serdang, Malaysia; 5 Dr. Boo Yang Liang, MRCP Hospital Enche’ Besar Hajjah Khalsom, Jalan Kota Tinggi, 86000 Kluang, Johor, Malaysia

**Keywords:** Acute coronary syndrome, Young adults, Malaysia, Ischemic heart disease

## Abstract

**Background and Objective:**

Acute coronary syndrome (ACS) is one of the leading cause of morbidity and mortality worldwide. It is relatively uncommon in young adults as compared to the older population. Our objective was to assess the prevalence, demographic distribution, and risk factors for acute coronary syndrome (ACS) in patients less than 45 years of age admitted to a Malaysian tertiary care centre.

**Methods:**

This is a cross-sectional, retrospective, and single centre study with random sampling of the patients admitted for ACS to hospital from January 2005 to December 2013. Data were collected and analyzed. Patients less than 45 years of age were compared with patients more than 45 years of age.

**Result:**

A total of 628 patients were included in the study and with the prevalence of young ACS was 6.1% and mean age of 39±6 years. All the young ACS patients were diagnosed with unstable angina and non-ST elevation myocardial infarction (NSTEMI). Tobacco smoking and family history of coronary artery disease (CAD) were more frequent in young ACS. 59.5% of the young ACS patients were smokers, while 37.8% and 51.4% of them were found to suffer from diabetes mellitus and hypertension respectively. Tobacco smoking, diabetes mellitus, and hypertension had shown significant association with the onset of young ACS (p ≤ 0.05).

**Conclusion:**

Three leading risk factors (tobacco smoking, diabetes mellitus, and hypertension) had been shown to be significantly associated with the onset of young ACS. Thus, it is important to identify this cohort and implement aggressive measures in tackling the risk factors in order to prevent or halt the development of coronary artery disease.

## INTRODUCTION

Ischemic heart disease (IHD) has been identified as one of the leading cause of death by World Health Organization (WHO) in 2012.^1^ In Malaysia, coronary artery disease (CAD) is one of the leading cause of mortality, accounts for 20-25% of all deaths in public hospitals.^2^ Acute coronary syndrome (ACS) is a clinical spectrum of ischemic heart disease ranging from unstable angina, non-ST segment elevation myocardial infarction (NSTEMI) to ST segment elevation myocardial infarction (STEMI). ACS among the young population is relatively low compared to older population.^3^ The prevalence of ACS among population less than 40 to 45 years of age ranged from 2 to 10% based on studies conducted from different countries around the world.^3^-^10^ Cardiovascular risk factors, such as smoking, hyperlipidemia, obesity, and family history of CAD, have been identified as more frequent among young ACS in these studies.^3^-^10^ At present, there is limited data on the prevalence and risk factors involving young ACS in Malaysia.

The aim of this study was to assess the prevalence and risk factors for ACS in population of 45 years of age. Thus, this study will provide the fundamental core for multi-centre studies to be carried out in Malaysia in the future.

## METHODS

### Subjects and data collection

This was a cross-sectional, single centre, retrospective study of young patients, aged less than 45 years old, being hospitalized with the diagnosis of ACS from the year 2005 to 2013. The simple random sampling method was utilized for sample selection. Data collection included factors that contribute to the prevalence of young ACS. Patient’s medical records were assessed, and demographic characteristic were recorded. Other clinical data such as the presence of co-morbidities (e.g. diabetes mellitus, hypertension, dyslipidemia), laboratory, and imaging reports were recorded as well. This study was approved by Medical Research and Ethics Committee (MREC) and Jawatankuasa Etika Universiti Putra Malaysia (JKEUPM).

### Statistical analysis

Statistical analysis was performed using IBM SPSS version 21.0. Descriptive statistics was used to describe the data. Categorical data was expressed as frequencies and percentages. For continuous data, means and standard deviations were presented. The association between the variables were analysed using Chi-Square Test of Independence. Statistical significance is defined as p-value < 0.05. Kolmogorov-Smirnov Test of Normality had been used to test the normality of a sample.

## RESULTS

A total of 628 patients were enrolled in this study. Table-I shows the demographic and baseline clinical characteristics of the patients. The association between age, risk factors and the onset of ACS are illustrated in Table II and III respectively. The prevalence of young ACS in this centre was 6.1% with the mean age of 39±6 years. All the young ACS patients were diagnosed with unstable angina and NSTEMI. Male (68.4%) were relatively more common in this young cohort compared to female (31.6%). Among the risk factors studied, tobacco smoking (59.5%) and family history of coronary artery disease (CAD) (29.7%) were more frequent in young ACS patients compared to older patients. Conversely, older patients had higher prevalence of diabetes mellitus (56.1%), hypertension (70.9%), and dyslipidemia (22.3%). Tobacco smoking, diabetes mellitus, and hypertension had shown significant association with the onset of young ACS (p ≤ 0.05).

**Table-I:** Distribution of demoghraphic among patients with acute coronary syndrome in the hospital.

		Frequency (n)	Percentage (%)
Age	Young ≤45	38	6.1
	Old >45	590	93.9
Gender	Male	450	71.7
	Female	178	28.3
Race	Malays	314	49.8
	Non Malays	316	50.2
Nationality	Malaysian	608	96.3
	Non Malaysian	20	3.2
Family History of CAD	Yes	129	20.5
	No	497	79.1
Smoking	Yes	270	43.0
	No	356	56.7
Diabetes Mellitus	Yes	345	54.9
	No	283	45.1
Hypertension	Yes	438	69.7
	No	190	30.3
Dyslipidemia	Yes	301	47.9
	No	323	51.4

**Table-II:** The association between age and young acute coronary syndrome.

Age	**Young Acute Coronary Syndrome (YACS)**				X^2^	P value

	Unstable Angina, STEMI NSTEMI					

	n	%	n	%		
Young (≤ 45)	38	100.0	0	0.0	11.286^a^	0.001
Old (> 45)	453	76.8	137	23.2		

**Table-III:** The association between the risk factors with the onset of young ACS.

Variables		Young Acute Coronary Syndrome (YACS)				X^2^	P value

		Yes		No			

		n	%	n	%		
Gender	Male	26	68.4	424	71.9	0.208^a^	0.648
	Female	12	31.6	166	28.1		
Race	Malays	22	57.9	291	49.3	1.049^a^	0.306
	Non Malays	16	42.1	299	50.7		
Nationality	Malaysian	34	89.5	574	97.3	NA	0.027
	Non Malaysian	4	10.5	16	2.7		
Smoking	Smokers	22	59.5	248	42.1	4.275^a^	0.039
	Non Smokers	15	40.5	341	57.9		
Family History	Yes	11	29.7	118	20.0	2.000^a^	0.157
	No	26	70.3	471	80.0		
Diabetes Mellitus	Yes	14	37.8	330	56.1	4.703^a^	0.030
	No	23	62.2	258	43.9		
Hypertension	Yes	19	51.4	417	70.9	6.318^a^	0.012
	No	18	48.6	171	29.1		
Dyslipidemia	Yes	6	16.2	137	23.3	1.000^a^	0.317
	No	31	83.8	450	76.7		

## DISCUSSION

In this study, 6.1% of patients with ACS were less than 45 years of age. This finding was consistent with prevalence rate in studies conducted from several countries, ranging from 2 to 10%.^3^-^10^ The mean age of young ACS was 39±6 years. As compared to older population, the frequency of ACS was much lower in the younger population (6.1% vs. 93.9%). The older population had a higer frequency of multiple risk factors such as diabetes mellitus, hypertension, and dyslipidemia. This increased the risk of developing cardiovascular diseases.

This study has shown that ACS in young patients occurred predominantly in men as compared to women. This observation was consistent with finding from previous reports. It has been postulated that in premenopausal women, estrogen plays an important role with its cardioprotective effect. Estrogen has the ability to lower the low density lipoprotein (LDL) and inhibits platelets aggregation.^11^ Thus, lower the risk of ACS among women.

With regards to ethnic differences, young ACS was more prevalent among the Malays (49.8%), followed by Indians (24.4%), Chinese (21.8%), and other races (4.1%). Of a total population of 28.3 million in 2010, Malays made up 55%, Chinese 24%, Indians 7% and other Indigenous people 13%.^12^ However, the proportion of the three main ethnic groups was not similar to the distribution of Malaysian national population in our study as evidenced by high prevalence rate among the Indians. Hughes et al. had reported higher mortality of ACS among Indians in Singapore in their papers published in 1990.^13^ Similarly, Chew et al. in 2011 reported a high prevalence of IHD amongst Indians complicated with diabetes mellitus.^14^ This observation could be explained by higher prevalence of diabetes mellitus amongst Indians as reported in our National Health Morbidity Survey (NHMS) III in 2006.^15^

Tobacco smoking had been identified as one of the major risk factors that contributed to young ACS in several studies.^3^-^10^ Exposure to tobacco is found to cause endothelial cells damage, leading to endothelial dysfunction, and injury to the vascular intima via a complex pathway.^16^ Smoking behavior among the young adults had found to accelerate atherosclerosis compared to older population.^5^ Smoking was the predominant risk factor in young ACS patients with its prevalence of nearly 60% and a p-value of 0.039 in our study. Previous studies had reported a higher prevalence of smoking in young ACS ranging from 70 to 90% of the studied population.^3^-^10^ With nearly a quarter of adults in Malaysia is an active smoker, this finding is important as preventive measures, such as campaign and law enforcement need to be intensified, thus, reducing the incidence of ACS in our population.^17^

The family history of CAD is associated with increased risk of young ACS. It had been reported that the risk of developing first acute myocardial infarction in patients with positive family history was more than a decade earlier compared to those without a family history of CAD.^18^ Several studies had reported the frequency of young ACS with family history ranged from 24 to 40%. Furthermore, there were genomic studies to suggest certain chromosomal abnormalies had contributed to the onset of ACS.^19^ This has strengthened the belief that family history is an important risk factor for CAD. In our study, only 29.7% of the young ACS presented with a positive family history of CAD, which was statistically insignificant (p-value = 0.157). We predicted that this might relate to missing data on family history of the hospital medical record system. Despite that, it remains one of the risk factors that may predict the onset of ACS in young populations.

Diabetes mellitus and hypertension are two well-established major risk factors for CAD. Diabetes is a metabolic disease with explicit complication on the coronary blood vessels by percipitating atherosclerosis.^20^ Hypertension will lead to plaque ruptured within the coronary arteries and causing thrombosis and occlusion of the circulation. Sympathetic hyperactivity in hypertensive patients also promotes the onset of ACS, coronary spasm, and coronary thrombosis.^21^ Our study had shown that older patients had a higher frequency of diabetes mellitus, hypertension, and dyslipidemia, as compared to younger patients. Despite that, nearly half the young ACS were hypertensive, and, at least, one-third were diabetic. These two risk factors were statistically significant in association with young ACS with p-value 0.030 and 0.012, respectively in this study. Lamk et al. had proposed poor diabetic control was responsible for the onset of young ACS despite low prevalence amongst the young populations.^22^ In Malaysia, NHMS IV in 2011 had illustrated marked increment in the prevalence of diabetes mellitus and slight increment in hypertension.^17^ This had signified the importance of lifestyles modification and optimization of treatment in reducing the cardiovascular outcome amongst the population.

Dyslipidemia is manifested by elevation of total cholesterol (TC), low density lipoprotein cholesterol (LDL-C), triglycerides (TG) with low levels of high density lipoprotein cholesterol (HDL- C).^23^ Only a small percentage of young ACS in our study suffered from dyslipidemia, and thus, statistically not significant (p-value = 0.317). In contrarary, studies by Schoenenberger et al. and Uranga et al. had shown a significant association between dyslipidemia and young onset ACS.^9^,^24^ Although this discrepency cannot be explained precisely, factors such as dietary and lifestyles may be involved. Despite that, the utilization of statin is well-proven as secondary prevention regardless of the TC and LDL-C level.

### Study limitation

The major limitation of our study is its observational and retrospective analysis. Thus, there were missing data from the registry which ultimately affect the study outcome. As this study only enrolled in-hospital patients from a single centre, this may not reflect the overall burden in the studied population. We suggest future studies to expand the studied area as well as include interventions and clinical outcome.

## CONCLUSION

Three leading risk factors (tobacco smoking, diabetes mellitus, and hypertension) had been shown to be significantly associated with the onset of young ACS. Thus, it is important to identify this cohort and implement aggressive measures in tackling the risk factors in order to prevent or halt the development of coronary artery disease.
